# Designing a new physical activity calorie equivalent food label and comparing its effect on caloric choices to that of the traffic light label among mothers: a mixed-method study

**DOI:** 10.3389/fpubh.2023.1280532

**Published:** 2023-11-14

**Authors:** Shirin Seyedhamzeh, Saharnaz Nedjat, Elham Shakibazadeh, Azam Doustmohammadian, Hedayat Hosseini, Asma Kazemi, Nafiseh Azizolahi, Maryam Chamary, Cain C. T. Clark, Ahmadreza Dorosty Motlagh

**Affiliations:** ^1^Department of Community Nutrition, School of Nutritional Sciences and Dietetics, Tehran University of Medical Sciences, Tehran, Iran; ^2^Students’ Scientific Research Center, Tehran University of Medical Sciences, Tehran, Iran; ^3^Department of Epidemiology and Biostatistics, School of Public Health, Tehran University of Medical Sciences, Tehran, Iran; ^4^Department of Health Education and Promotion, School of Public Health, Tehran University of Medical Sciences, Tehran, Iran; ^5^Gastrointestinal and Liver Diseases Research Center, Iran University of Medical Sciences, Tehran, Iran; ^6^Department of Food Sciences and Technology, National Nutrition and Food Technology Research Institute, Shahid Beheshti University of Medical Sciences, Tehran, Iran; ^7^Nutrition Research Center, Shiraz University of Medical Sciences, Shiraz, Iran; ^8^Centre for Intelligent Healthcare, Coventry University, Coventry, United Kingdom

**Keywords:** nutrition label, physical activity, TLL, obesity, PACE

## Abstract

**Objective:**

We designed a new type of ‘physical activity calorie equivalent’ (PACE) food label in Iran to compare its effect with that of the traffic light food label (TLL) on caloric choices.

**Design:**

Mixed-method study.

**Participants:**

Mothers of school children between the ages of 6–12 years.

**Setting:**

In the qualitative phase, 10 focus group discussions (FGDs) were conducted with various groups of mothers, and two FGDs were conducted with food science and nutrition experts to design a new PACE label. In the quantitative phase, 496 mothers were randomly assigned to five groups: (1) no nutrition label, (2) current TLL, (3) current TLL + educational brochure, (4) PACE label, and (5) PACE label + brochure. Samples of dairy products, beverages, cakes, and biscuits were presented. ANOVA and multiple linear regressions were applied to examine the association between label types and calories of the selected products as our main outcome.

**Results:**

The mothers’ perspectives were classified into two sub-themes, the PACE label’s facilitators and barriers. The new PACE label’s characteristics were divided into two subcategories: (a) appearance, and (b) nutritional information, including 14 codes. In the quantitative section, mean calories of the selected foods were lowest in the TLL + brochure group (831.77 kcal; 95% CI: 794.23–869.32), and highest in the PACE label group (971.61; 95% CI: 926.37–1016.84).

**Conclusion:**

The new PACE label was a combination of PACE, TLL, and warning labels. It did not significantly affect lower caloric choice, however, the TLL + brochure option was effective in choosing foods with fewer calories.

**Clinical trial registration**: The study was registered in the Iranian Registry of Clinical Trials 23 (IRCT20181002041201N1).

## 1. Introduction

The increasing prevalence of obesity has been, and remains, a major global challenge, where rapid changes in dietary and food behaviors –and as a consequence, excess energy intake- are the main causes of this rising trend (1). According to recent studies, the rates of mortality caused by non-communicable diseases (NCD) are higher in low and middle-income countries (2, 3).

Energy imbalances and physical inactivity in children and adolescents are of particular concern (4). Member states of the World Health Organization (WHO) agreed to reduce physical inactivity by 10% by 2025, and operational policies are underway in 56% of these states (5); Iran also aims to reduce its physical inactivity by 20% by 2025 (6). Therefore, in order to mitigate this harmful trend, an effective policy must be developed to target both unhealthy diets and physical inactivity (4). However, these policies may be implemented differently in various countries or in different populations, e.g., children (7, 8).

Nutrition labels, taxes, and subsidies on healthy foods are examples of policy interventions that can target public behavior and the environment. Food labeling, in particular, has attracted the interest of researchers and policymakers, since it is considered as a nudging strategy that can encourage healthier eating (9). However, the main limitation of food labels’ effectiveness is the difficulty in understanding their information (10). Various types of numerical and interpretive labels, such as nutrition fact, traffic light, and star rating labels exist in different countries (11–13). However, there is no consensus among investigators on the impact of nutrition labels on food behavior (14–16).

Traffic light labels (TLL) were first invented by non-governmental organizations in the United Kingdom in the early 1990s (17). After multiple researches, it was agreed to announce the amounts of four nutrients including, sugar, sodium, fat and saturated fatty acids using green, yellow and red colors, the colors of traffic lights. Each of these colors indicates low, medium, and high, respectively (17). Numerous studies have assessed the impact of TLL on healthier food selection (18–21); most of them have indicated TLL as an effective tool in healthier food choices. The results of a systematic review and meta-analysis study showed that TLL was marginally more effective than the Guideline Daily Amount (GDA) and other food labels in increasing the selection of healthier options (22). An important point about the studies on TLL effectiveness is that many of these studies have been done on food products such as pizza, sandwiches, popcorn and breakfast cereals.

Recently, a new type of label that takes physical activity into consideration has been examined in different studies in high-income countries (23–29) that shows the amount of physical activity needed to ‘burn off’ calories. Thus, this label employs a multi-disciplinary strategy that combines both caloric content and physical activity. Therefore, it can help to improve public awareness about the importance of physical activity in reducing the trend of obesity; it can also lead to a healthier lifestyle among the general population (30, 31) A recent systematic review and meta-analysis of randomized controlled trials and experimental studies revealed that, compared to other food labels, using physical activity calorie equivalent (PACE) labeling can be helpful in consuming significantly fewer calories by the public (32). Nonetheless, perceptions about PACE labels have not been sufficiently investigated, and the existing literature has been restricted to high-income countries (33).

Although food labels have been used in Iran for many years, studies show that consumers’ usage rates are low (34, 35). Among the reasons behind food labels’ inefficacy are their incomprehensibility and the high load of information presented in them. In recent years, the use of traffic light labels in packaging products has become mandatory in Iran, but many challenges have been raised in the use and interpretation of its colors by consumers and experts. To this end, for the first time in Iran, we sought to design a food label that includes the level of physical activity, by considering the viewpoints of mothers, who overwhelmingly represent Iranian households, alongside food science and nutrition experts (PhD graduates of nutrition and food industry sciences). By doing so, we intended to convey the most important information (in addition to the amount of physical activity needed to burn off calories), as well as the best appearance features to consumers in the shortest possible time, and to encourage them toward choices with fewer calories. To our knowledge, this is the first randomized study to compare the effect of PACE and TLL on packaged food products in Iran.

## 2. Methods

This study was part of a larger mixed method research in which the strengths, weaknesses, and strategies of improving the use of nutrition labels were discussed (36). Subsequently, a new PACE label was designed based on findings from a qualitative study, and examined in a trial to assess its impact on food choices (quantitative phase). The study’s protocol has been published elsewhere, where the method has been discussed in detail (37). Specifically, we investigated whether a label that has been designed based on recommendations made by mothers (household representatives) and professionals (scientific representatives), could guide consumers to healthier food choices in terms of calories compared to TLL as a mandatory label on food packaging in Iran.

For the qualitative phase, the participants were informed of the research objectives before the interviews. Furthermore, participants were assured that the information would be used only for research purposes, and would not be accessible to those who were not in the research team. For the quantitative phase, written informed consent was obtained from all participants.

### 2.1. Qualitative phase

**Figure 1:**
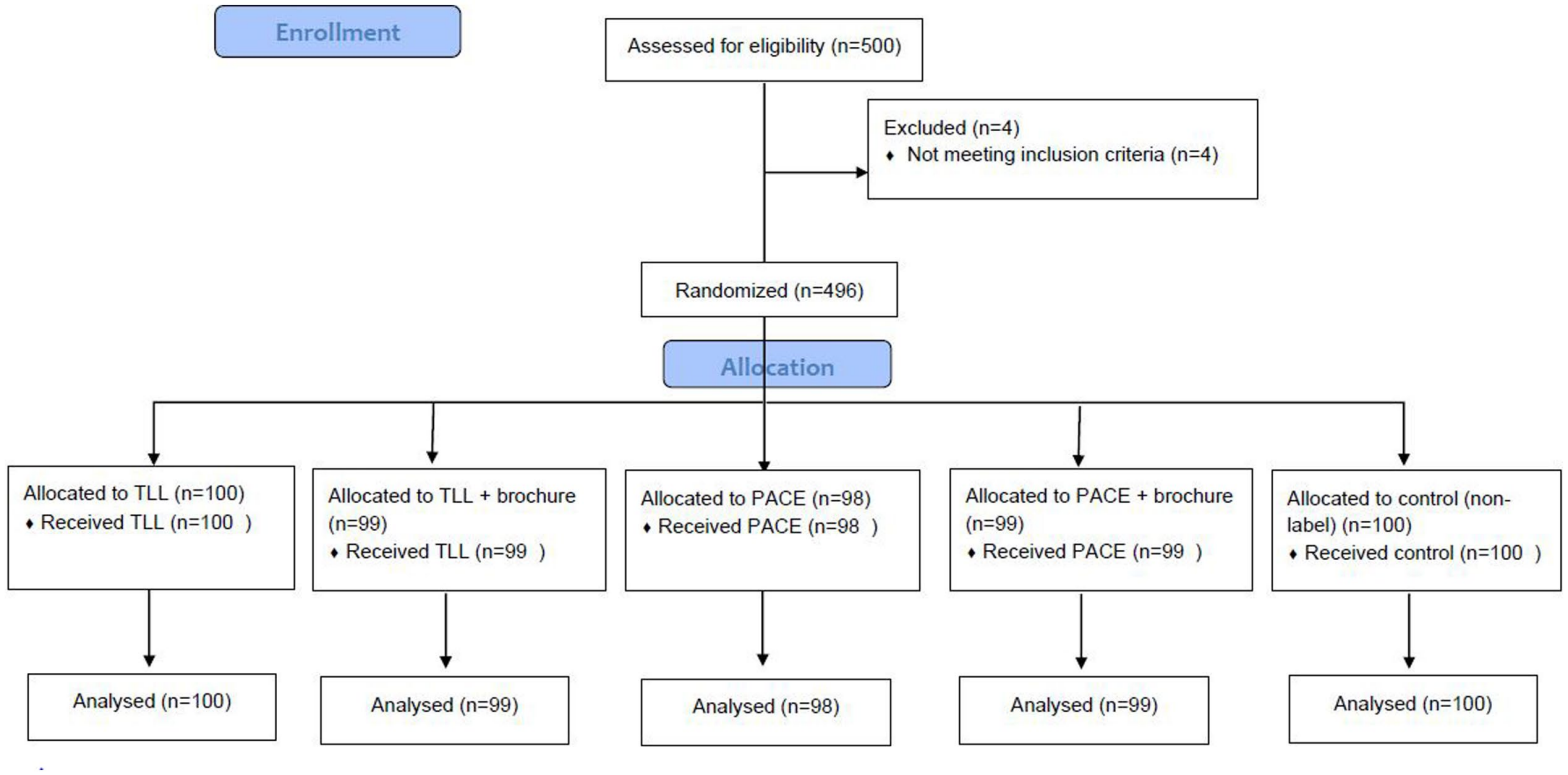
Study flowchart.

A flowchart showing the study’s processes is presented in Figure 1. All the sessions were conducted with an investigator (first author, who was a PhD graduate of ‘Food and Nutrition Policy’) and a note taker trained in qualitative research, using a questionnaire guide (Supplementary File 1). Moreover, all the focus group discussions (FGDs) were transcribed. The Consolidated Criteria for Reporting Research (COREQ) (38) and the Standards for Reporting Qualitative Research (SRQR) (39) were followed in the conducting of this research.

#### 2.1.1. FGDs with mothers as household representatives

Sixty-three mothers participated in our study. The mothers’ general characteristics have been presented in Supplementary File 2: Table S1. We chose mothers because women are responsible for the purchase and preparation of foods in most Iranian households (40–42).

Ten FGDs were conducted with mothers of school children aged 6–12 years in ten primary schools from southern, northern, eastern, western, and central Tehran. Two primary schools were randomly selected from each district. This way, we achieved homogenous sampling with respect to the participants’ socioeconomic status. Primary school children’s mothers were chosen due to the importance of childhood obesity and their role as gatekeepers and choosing food. Primary schools in Iran include 6–12-year-old children. As mentioned earlier, 2 schools were randomly selected from the northern, eastern, western, central and southern areas of Tehran. All participants were literate and willing to participate. Mothers who were nutritionists or food technologists were excluded from the study; however, there were no limitations on their age. Mothers were invited to the respective schools by phone, and we continued recruitment until at least 6 mothers –who met our selection criteria- agreed to participate. All sessions were recorded with a voice recorder, after obtaining the participants’ consent. FGDs consisted of two parts: in the first part, the existing labels were discussed, and in the second part, the mothers were asked, for an average of 75 min, about the new PACE label. The researcher used a guide for asking questions (Supplementary File 1: Table S2). The concept of a PACE label was introduced to the mothers through products such as cakes with different calories, and as these products had different amounts of calories, the suggested physical activity for each label was different. Several initial layouts of the PACE labels were designed by a graphic designer based on the mothers’ points of view for further discussion in the experts’ FGDs. Interviews continued until data saturation was reached.

#### 2.1.2. FGDs with experts

Two FGDs were held with ‘food science and nutrition experts’ (hereinafter referred to as ‘FSN experts’ for the sake of brevity). For this group, the sampling method was purposeful and FSN experts from two universities of Tehran participated in the FGDs. First, official letters were sent to the Heads of Departments of Nutritional Sciences and Dietetics and the study objective was explained. Then, a day was coordinated to hold the FGD with the aforementioned professionals. The FGDs with FSN experts were conducted with 6 PhD graduates of nutrition and 8 PhD graduates of food sciences from universities in Tehran.

The mothers’ expectations regarding the PACE labels were discussed and the FSN experts’ comments were collected in two separate FGDs. The average duration of each FGD was about 1 h. The labels were then amended upon consensus among the researchers and sent to the mothers to outline the final format of the PACE labels. Most of the changes the mothers requested pertained to the appearance of the new PACE label. Ultimately, the final versions of the new labels were designed.

#### 2.1.3. Label printing

After confirming the label image for print, we had to determine the size and definitions on each label. For this purpose, the information on the purchased products, such as definitions of serving, weight of each serving, total weight of the product, and the label size were entered into Excel 2010 software, and understandable serving, label size, energy, sugar, salt, fat, physical activity level per minute, required number of labels, and color were added to the information of each product. According to the size of various food labels on the products, the 2 × 3 cm size was applicable to all dairy products, cakes, biscuits and sugar-sweetened beverages. After print, the readability of the labels was confirmed by the researchers.

#### 2.1.4. Qualitative phase data analysis

The conventional content qualitative analysis approach was applied to determine the main themes (43). This method is used when there is insufficient evidence and inaccurate data on a specific topic or phenomenon.

All the FGDs were transcribed verbatim and typed in Microsoft Word. Notes taken by the note taker were added to the transcription to ensure accuracy and clarity of any instances which might not have been recognized by the transcriber. The MAXQDA 10 software was used to help qualitatively analyze the interviews. We identified the themes after systematically coding the data by reading and re-reading (43). In this technique, the text was read like a novel and key words or sentences that addressed the concepts and perspectives were marked. Thereafter, the text was re-read several times and codes relevant to one or several topics were recorded. Similar codes were then presented as themes. After reaching consensus, investigators developed a codebook with detailed transcriptions. To ensure validity and reliability, after the initial analysis of the interviews, approximately half of them were sent to the interviewees and were analyzed after their approval was obtained. Upon analyzing the transcriptions, approximately 40% of them were sent to the co-investigator and re-examined for consistency of coding (inter-coder agreement: 91%). The analysis results were approved by a quality-assurance supervisor.

### 2.2. Quantitative phase

Similar to the qualitative phase, the participants were mothers of 6-12-year-old schoolchildren. Ten schools were randomly selected from the same regions used in the qualitative phase, but the schools were different this time, so the mothers would be blinded toward the study goal regarding their caloric choices. The inclusion criteria were the same as those in the qualitative phase.

#### 2.2.1. Sample size calculation and randomization

Using G*Power software, sample size was calculated based on caloric choices –which is the primary outcome of our study. A sample size of 481 was calculated considering the expected effect size of 0.15, α = 0.05, and power = 90% (44). To account for potential attrition, a sample size of 500 was estimated (100 in each arm).

We applied stratified block randomization, using STATA software, where stratums were schools, and each stratum contained five blocks of ten.

#### 2.2.2. Study procedure

**Figure 2:**
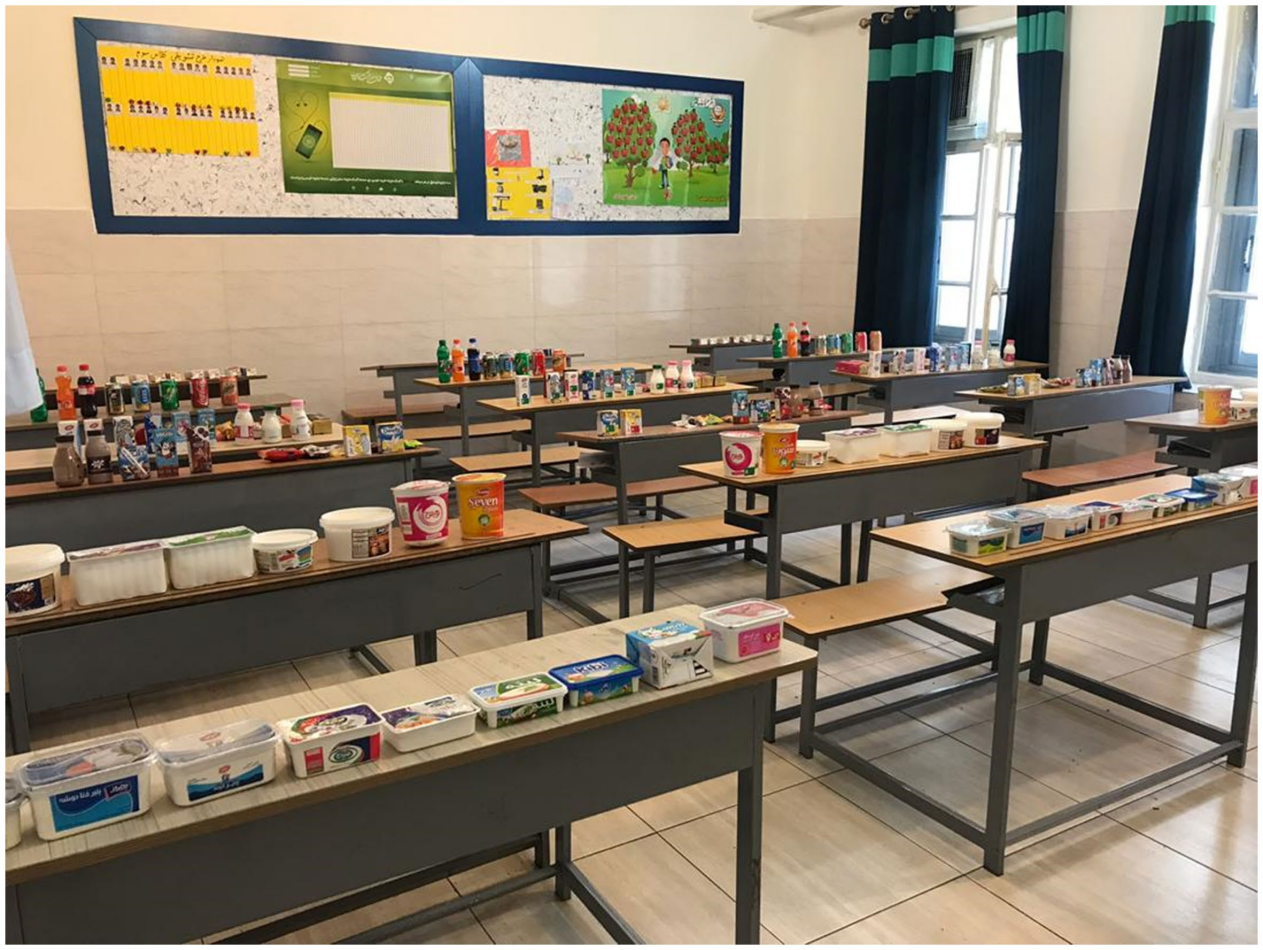
The food products used for the study.

**Figure 3:**
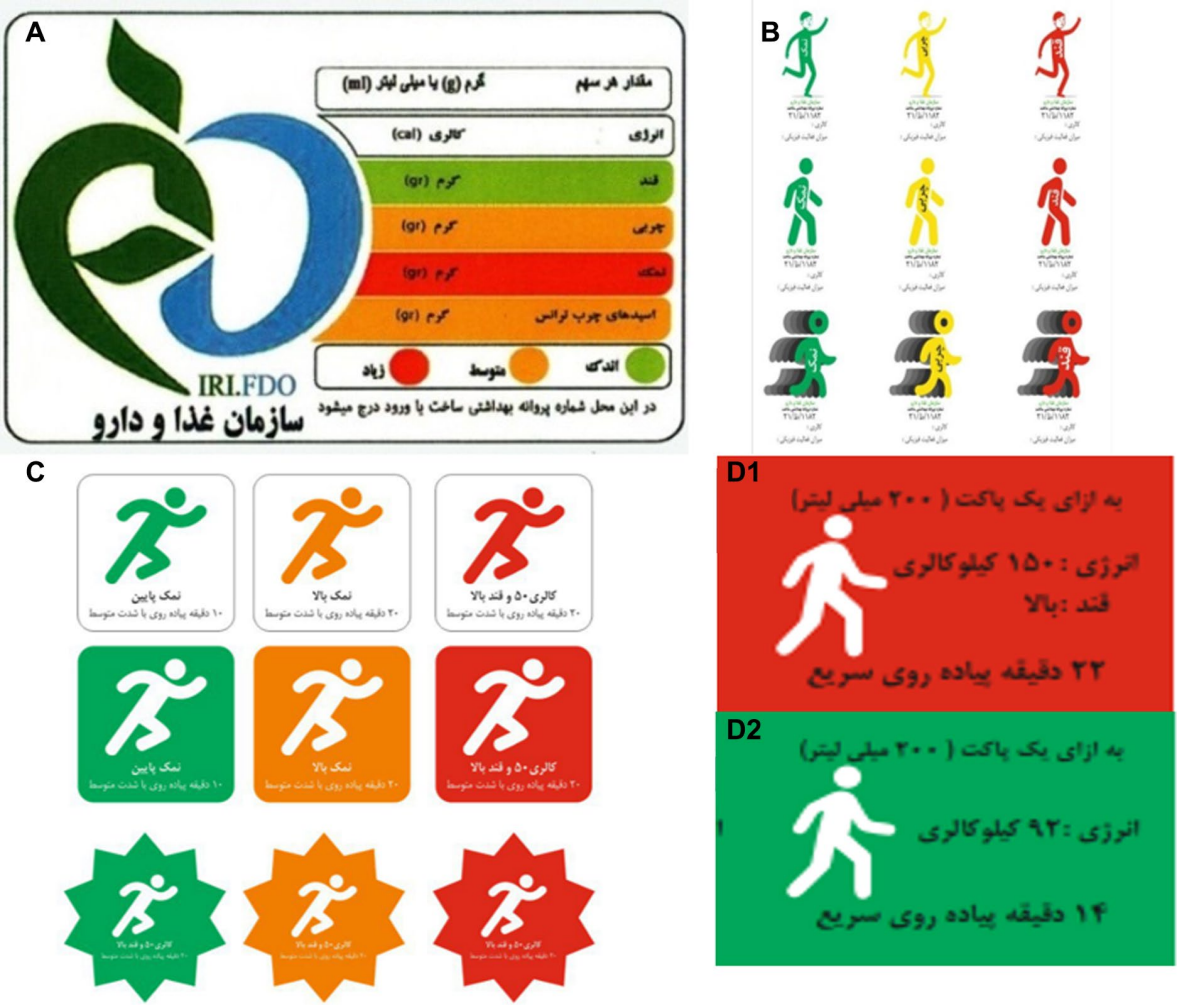
The food products used for the study. **(A)** Shows energy (Cal), Serving size (g)/(mL), sugar (g), fat (g), salt (g), *trans* fatty acids (g) (green: low, amber: medium, red: high). **(B)** The first PACE label designed: first row represents the salt, fat and sugar content in different colors; energy (Cal), the amount of physical activity. Second and third rows are similar to the first row but different in image design due to the participants’ viewpoints (green: low, amber: medium, red: high). **(C)** The second PACE label designed with the clause “low amount and high amount of salt/fat/sugar,” energy and the duration of physical activity required to burn off calories (green: low, amber: medium, red: high). **(D1)** One packet (200 mL), energy: 150 Kcal, sugar: high (red), 22 min brisk walking. **(D2)** One packet (200 mL), energy: 92 Kcal (green), 14 min brisk walking.

First, a pilot study was conducted in one school (on 50 mothers) to assess the likely problems, practicality, and feasibility of the study. In the second phase, mothers were randomized into five arms, including the traffic light label (TLL)- which is currently mandatory in Iran-, TLL plus the educational brochure (Supplementary File 2), PACE, PACE plus the educational brochure, and without the label arm, using a randomization list. PACE was initially designed by a graphic designer and changes were made based on recommendations made by mothers and specialists. The demographic questionnaire and international physical activity questionnaire (IPAQ) were filled before the participants were offered different food choices (Supplementary File 3). The Consolidated Standards of Reporting Trials (CONSORT) flow diagram is presented in Figure 2. The participants were blinded to the primary objective of the study and were told the objective was to identify “factors affecting a family’s food choices and their impacts on children’s nutritional status and anthropometrics.” Food selection was performed among 42 dairy products, 29 beverages, and 38 cakes and cookies (Figure 3). Each participant was allowed to select 8 food products from 8 food groups including 1-milk, 2-flavored milk, 3-cheese, 4- yogurt, 5-juice, 6-carbonated drinks, 7-cakes, and 8-biscuits and wafers. In each group, food products had different calories and so there were options with fewer calories. The food products were provided from a chain store and were from well-known brands. Participants were requested to suppose they were in a supermarket and to choose their preferred products among the provided foods. They were also requested to read a brochure before they began to purchase the products.

#### 2.2.3. Quantitative phase data analysis

Continuous variables were analyzed by analysis of variance (ANOVA) (comparing mean calories of selected foods between the groups), and categorical variables by chi-square tests [comparing general characteristics including marital status, education, economic status, satiety status, BMI (normal/overweight/obese), and physical activity]. Linear regression was used to assess the relationship between the types of labels and the primary outcome, which was the caloric choice of select products. To adjust for the effect of potential confounders (age, economic status, education, job, BMI, and physical activity), multivariable linear regression was used. The potential confounders were assessed using a demographic questionnaire (Supplementary File 3). All tests were conducted using STATA 16, and type I error was considered as 0.05.

## 3. Results

### 3.1. Qualitative phase: designing a new PACE label

In this section, two main themes were defined. First, the mothers’ perspectives on PACE were examined, and then the new label’s expectations were drafted upon taking into account the viewpoints of mothers and FSN experts. The aforementioned are presented in Supplementary File 1: Table S3.

#### 3.1.1. Mothers’ perspectives as household representatives

Mothers’ perspectives were classified into two sub-themes, the physical activity label’s facilitators and barriers. The facilitators were categorized into four codes: 1- healthier food choices, 2-easy to understand, 3-suitable solution, 4-creating incentives to lose weight and be physically active. The barriers were categorized into the following four codes: 1-no impact on level of activity, 2-lack of significance, 3-economic problems, 4-lack of time.

##### 3.1.1.1. Facilitators

Most mothers’ perspectives on PACE labeling were positive.

*“It’s very good to know how many calories you are receiving and how much activity you must have to burn them off”* (35-year-old mother).

The most important facilitator of the PACE label, according to the mothers, was choosing healthier foods. Some mothers believed that such a label did not require any specialist knowledge of nutrition. Furthermore, since the concept of physical activity –such as walking- is more comprehensible than calories, it can be easier to understand for the general public. Others stated that physical activity could be a suitable solution for certain food items of interest. If weight reduction was an important consideration for the individual, it could motivate them to increase their physical activities.

##### 3.1.1.2. Barriers

Economic problems were mentioned as a major barrier for using the label. However, this was not mentioned for the PACE label alone. Lack of time was also mentioned as a probable barrier. Some mothers believed that PACE would change their food choices but not their physical activity habits, thus lack of impact on level of activity was another mentioned barrier. Based on their opinions, PACE primarily affected their food choices, but physical activity would gradually change over time, or be used more often by athletes or people actively concerned about their fitness.

Very few mothers (two) said that even if the label was very simple, it would still have no significant impact on their food choices. Therefore, even if the label were simple in its design or appearance, it still may not affect their choice of food selection.

Not all mothers agreed on how these labels would affect their children’s choices. Some mothers were certain that they would improve their children’s food behavior, while others considered them as ineffective. The reasons given for these dissimilar opinions were, the children or their family habits and, the influence of their peers. Indeed, some participants were certain that this type of label could change their children and their own behavior toward eating foods, leading to healthier food choices in the family.

*“When a child sees that his or her parents care about everything, that child too, will learn to do so.”* (30-year-old mother).

#### 3.1.2. Mothers’ expectations of the new PACE label

The participants unanimously agreed that graphics were more understandable than digits. They also said that walking was the most feasible form of physical activity, as compared to other exercises, such as swimming. Colorful labels were highly recommended, especially for children. In addition, most of the mothers believed that using both PACE and TLL (Figure 3A) would be more effective. Another point raised by the mothers was the location of the label. They said that nutrition labels were mostly positioned at the back of packages, at inappropriate locations, and that they were mostly illegible; they had to spend a lot of time finding and reading the labels, thus, they might be overlooked. Many suggested that the nutrition label should be near the production/expiry date, where it would be noticed, or near the brand name, on the front of the package. The participants believed “walking duration” in minutes was a more appropriate term than “walking distance” in kilometers. In their opinion, every single nutritional fact was important, such as the general and detailed information about fat, sugar, salt, vitamins and minerals, calorie content and understandable portion size. However, this may not be possible, because printing all of this data in the form of colorful graphics can affect the labels’ legibility.

Taking the mothers’ viewpoints into consideration, with the help of our graphic designer we designed three different PACE labels by the end of the FGDs (Figure 3B). We simplified the images by excluding certain variables, such as age and gender, to produce an applicable label. In order to convert calories into physical activity, walking at 5.6 km/h, an average energy consumption of 6.7 kcal/min, and an average weight of 70 kg were considered for the calculations. For example, a pack of cake with 228 calories required 34 min brisk walking to burn off. The investigators collectively agreed on the second image; however, the walking image was changed to brisk walking in order to better represent the information (Figure 3C).

#### 3.1.3. Experts’ expectations of the new PACE label

The food science and nutrition (FSN) experts approved the graphic design of the new label, and believed it to be easily understood by the public. However, some of them suggested the PACE information to be included in the nutrition fact table.

All the experts believed that using a single color instead of three would simplify matters for the consumer. They believed that consumers ought to be provided with vital, yet concise information. Drawing from our results, we decided to use one of the green or red colors on the labels to highlight the high amount of fat, sugar, and/or salt. Based on Iran’s Food and Drug Administration guidelines (45), if any of the fat, sodium, or sugar contents of a product are in the red zone (high), the red label should be used (the cutoff points used for allocating a color to a food product based on the values of fat, sugar and sodium have been listed in Supplementary File 1: Tables S4A,B). In addition, the clause “high in fat/sugar/sodium,” along with calorie content, serving size, and recommended physical activity will be included in the label. However, if the abovementioned content levels are in the amber (medium) or green (low) zones, only the green label will be used along with calorie content, serving size, and recommended physical activity. Regardless of the label’s color, the minutes of physical activity required to burn off the calories must be provided. Thus, the second format of the picture (Figure 3B) was selected. Finally, the new labels were designed in red and green. The red label provides extra information on the high amount of fat and/or salt and/or sugar. After taking into account the FSN experts’ opinions, and a consensus among the investigators, the labels were sent to the mothers. They were asked to select their preferred label(s) and/or make changes that would improve their food choices. They believed that walking was a more popular activity, so people may find the information more applicable to their lives.

“*The picture suggests running instead of walking, which is a limitation and may not be possible for many women in Iran.*” (45-year-old mother).

Thus, the figurative label was slightly amended based on the mothers’ opinions (Figure 3D) and the new PACE label was designed after considering all the stakeholders’ viewpoints.

### 3.2. Quantitative phase: assessing the effect of the new PACE and TLL

The general characteristics of participants of different groups are presented in Table 1. A total of 496 mothers participated in the study. There were significant differences between the groups in terms of the participants’ age, education, and physical activity. Moreover, the blocks were matched according to gender and location.

**Table 1:** General characteristics of participants in the quantitative phase.

Variables	Total *N* (%)^a^	Groups					*p*-value^b^
		Without label *N* (%)	TLL *N* (%)	TLL + brochure *N* (%)	PACE *N* (%)	PACE + brochure *N* (%)	
Participants	496 (100)	100 (20.2)	100 (20.2)	99 (20)	98 (19.8)	99 (20)	
Marital status (married)	480 (96.86)	97 (97)	98 (98)	94 (94.9)	95 (96.9)	96 (97)	0.30
Education (academic education)	189 (38.1)	53 (53)	29 (29)	19 (19.2)	50 (51)	38 (38.4)	<0.001
Family members ≤4	436 (87.9)	84 (84)	90 (90)	83 (82.3)	91 (82.6)	89 (89.9)	0.06
**BMI (kg/m** ^**2**^ **)** ^d^							
Normal (18–24.9)	154 (31.17)	30 (30)	32 (32)	36 (36.7)	32 (32.7)	24 (24.5)	0.65
Overweight (25–29.9)	211 (42.71)	44 (44)	40 (40)	37 (378)	37 (37.8)	49 (50)	
Obese (>30)	129 (26.11)	26 (26)	28 (28)	29 (29.6)	29 (29.6)	25 (25.5)	
**Economic status based on assets**							
Highest	100 (20.2)	19 (19)	23 (23)	26 (26.3)	19 (19.4)	13 (13.1)	0.37
High	99 (20)	22 (22)	23 (23)	24 (24.2)	17 (17.3)	13 (13.1)	
Moderate	99 (19.4)	19 (19)	20 (20)	14 (14.1)	22 (22.4)	24 (24.2)	
Low	100 (20.2)	23 (23)	15 (15)	17 (17.2)	19 (19.4)	26 (26.30)	
Lowest	98 (19.8)	17 (17)	19 (19)	23 (23.2)	21 (21.4)	23 (23.2)	
**Occupation**							
Housewife	395 (79.64)	83 (83)	80 (80)	83 (83.8)	75 (76.5)	74 (74.7)	0.08
Worker	6 (1.21)	1 (1)	1 (1)	3 (3)	1 (1)	–	
Farmer	14 (2.82)	3 (3)	4 (4)	–	2 (2)	5 (5.1)	
Employed	14 (2.82)	4 (4)	1 (1)	–	6 (6.1)	3 (3)	
Administrative staff	19 (3.83)	2 (2)	6 (6)	6 (6.1)	4 (4.1)	1 (1)	
Retired	2 (0.4)	1 (1)	–	1 (1)	–	–	
Self-employed	45 (9.7)	6 (6)	7 (7)	6 (6.1)	10 (10.2)	16 (16.2)	
**Satiety status using visual analog scale**							
Very hungry	35 (7.06)	11 (11)	8 (8)	6 (6.1)	6 (6.1)	4 (4)	0.8
Quite hungry	72 (14.52)	11 (11)	17 (17)	18 (18.2)	13 (13.3)	13 (13.1)	
Not hungry or full	135 (27.22)	24 (24)	28 (28)	28 (28.3)	24 (24.5)	31 (31.3)	
Quite full	119 (23.92)	25 (25)	22 (22)	20 (20.2)	26 (26.5)	26 (26.2)	
Full	134 (27.02)	29 (29)	27 (27)	25 (25.3)	29 (29.6)	25 (25.2)	
**Physical activity score (ME/min/weak)** ^**c**^							
Light	344 (71.67)	80 (87.9)	71 (74.7)	69 (71.1)	61 (62.6)	63 (63.6)	0.007
Moderate	122 (25.42)	11 (12.1)	22 (23.2)	25 (25.8)	33 (33.7)	31 (31.3)	
Vigorous	14 (2.92)	-	2 (2.1)	3 (3.1)	4 (4.1)	5 (5.1)	
Age, years [Mean (SD)]	38.5 (6.87)	38.2 (4.82)	36.77 (4.86)	36.87 (5.63)	39.5 (4.84)	40.80 (13.10)	0.002

^a^The values are numbers (percentages) for all variables except for ‘age’, where the values are means (standard deviations). ^b^Values were obtained from one-way analysis of variance for quantitative variables and chi-square test for qualitative variables. ^c^Physical activity was assessed using the International Physical Activity Questionnaire (IPAQ). ^d^BMI: body mass index; ME: metabolic equivalent; PACE: physical activity calorie equivalent; TLL: traffic light label.

#### 3.2.1. Calories of selected foods

The mean calories of all the selected foods, based on the food groups, including dairy, sugar-sweetened beverages, cakes, and cookies have been listed in Table 2. The calories of the selected foods ranged from 334 to 1,425 kcal. The PACE label did not have a significant effect on choosing products with fewer calories compared to other groups. The total calories of the foods selected in the TLL + brochure group were significantly fewer compared to the other groups. Furthermore, the calories of dairy, sugar-sweetened beverages, cakes, and biscuits in the TLL + brochure were also lower than the others; however, the difference was statistically significant only for cakes and biscuits.

**Table 2:** Mean calories (kcal) and 95% confidence intervals of all the selected foods, based on the food groups, including dairy, sugar-sweetened beverages, cakes and biscuits.

Food products	Without the label (*n* = 100)	TLL (*n* = 100)	TLL + brochure (*n* = 99)	PACE (*n* = 98)^a^	PACE + brochure (*n* = 99)	*p-*value
All products	918.21 (877.367,959.05)	921.29 (880.16, 962.41)	831.77 (794.23, 869.32)	971.61 (926.37,1016.84)	916.73 (875.19, 958.28)	0.0001
Dairy	508.69 (478.34, 539.03)	493.2 (462.95, 524.44)	475.51 (449.01, 502.01)	531.1 (496.70, 565.49)	505.68 (473.59, 537.77)	0.15
Sugar-Sweetened beverages	109.65 (94.02, 125.27)	118.63 (104.13, 133.12)	99.96 (82.83, 117.10)	112.55 (96.90, 128.19)	108.13 (92.41, 123.85)	0.57
Cakes and biscuits	299.87 (277.01, 322.72)	309.46 (284.27, 334.64)	256.29 (10.91) (234.84, 277.74)	327.95 (300.01, 355.90)	302.91 (276.03, 329.80)	0.002

The mean calories of all food products were significantly fewer in TLL + brochure than in the other groups (without label, TLL, PACE, PACE + brochure). For the food groups, the calories of cakes and biscuits were significantly fewer in the TLL + brochure compared to the other groups. No significant relationship was found between the groups for other food groups. The reported values are means (standard deviations). *P* value was obtained using the ANOVA method. ^a^PACE: physical activity calorie equivalent; TLL: traffic light label.

Comparison of the intervention groups with the control group indicated that after adjusting for the confounders, only the TLL + brochure group’s selected calories were significantly lower than the control group (caloric diff = −76 Kcal; *p* = 0.01). Conversely, the PACE label’s calories were significantly higher than the control group’s (caloric diff = 61 kcal; *p* = 0.04). The differences between TLL as well as PACE+brochure and the control groups were insignificant (Table 3).

**Table 3:** Comparing the effects of PACE and TLL on calories of selected foods – bearing in mind the confounders’^1^ effects.

Comparisons	β Coefficients (95% CI)	*p-*value
Calorie intake (kcal, PACE /TL)	23.31 (−9.28, 55.91)	0.16
Mothers’ age (year)	0.91 (−5.66, 7.50)	0.78
Economic status based on assets^2^	−8.85 (−30.76, 13.06)	0.42
Education level (academic, non-academic)^3^	16.13 (−86.69, 118.96)	0.75
Occupation (employed, housewife)	55.80 (−21.33, 132.93)	0.15
BMI^4^		
Normal	Ref	
Overweight	8.12 (−66.32, 82.57)	0.83
Obese	114.27 (34.18, 194.36)	0.005
Physical activity^5^	16.24 (−42.06, 74.55)	0.58

^1^The confounders were age, economic status, education, occupation, BMI, and physical activity. ^2^Assets were categorized into quintiles from lowest to highest (The reference category was the lowest economic status). ^3^The reference category was non-academic education. ^4^Physical activity was categorized into tertiles (light, moderate, vigorous). ^5^Multivariable regression was used to compare the effect of the PACE label to TLL on the calories of selected foods. PACE: physical activity calorie equivalent; TLL: traffic light label.

With regards to the confounders’ effects, each year increase in maternal age was associated with a decrease of 1.41 kcal in the selected calories, however it was not statistically significant. Improvement of the economic status was associated with a decrease in the selected calories by 2 kcal, but it was not significant. A rise in the education level yeilded an insignificant decrease of 10 kcal in the seclected calories. Employed mothers chose about 38 kcal more than housewives, but it was not significant. In obese individuals, each unit increase in BMI yeilded an increase of 104 kcal in the selected calories, and was statistically significant (*p* = 0.001). Finally, each metabolic equivalent/min/week increase in physical activity level was associated with a non-significant decrease of about 13 kcal in the selected calories (*p* = 0.48) (Supplementary File 1: Table S5).

After adjusting for the other confounders (age, economic status, education, BMI, and physical activity), only BMI was significantly effective, such that obese individuals selected more calories (~114 Kcal).

## 4. Discussion

### 4.1. Qualitative phase

We designed a new label which is a combination of the traffic light, warning (46), and physical activity labels (47). It provided information about caloric content, serving size, and recommended physical activity with the clause *“high in* sugar/salt/fat.” As a result, the consumer will see either one of the green or red colors, and will be warned of the potentially harmful content/s. Therefore, this type of label can easily be understood, regardless of the consumer’s level of education. The color amber, which represents TLL’s medium level, was omitted to make its printing easier for manufacturers.

The PACE label was first designed by Swartz et al. (47) and was examined in various studies on a variety of foods, such as fast foods (23, 24, 27), snacks, and beverages (26, 27, 48) to discern its effectiveness. However, these studies’ findings were inconsistent (49–51).

In one study, a warning label was assessed for certain products that were high in sugar, sodium, and saturated fats (52, 53). The warning label was designed to prevent the rising trend of obesity; however, it only provided details about the high amounts of nutrients that had adverse effects on consumers’ health using the color black, which is associated with reduced perceptions of food healthiness. In our new label, not only are high amounts of the abovementioned nutrients presented, but consumers are also warned about the unhealthiness of the nutrients’ levels by presenting them in red color. Further information, such as caloric content, serving size, and recommended physical activity help provide customers with sufficient details to decide whether or not to purchase a product.

The findings of our study were concordant with the studies in which environmental and individual factors were assessed in menu labeling utilization. In Schindler et al.’s study, the most frequently cited barriers to menu label utilization were price and time constraints (54). The result of another study showed that parents’ decisions on what fast food items to order for their children might be affected by PACE labeling. Thus, this labeling could encourage them to get their children to exercise (27).

### 4.2. Quantitative phase

The PACE label failed to influence food choices in terms of caloric content. The lowest calories of the foods selected were seen in the TLL + brochure group in the pairwise comparison of the intervention vs. control group. The calories of the selected foods in the TLL+ brochure were 76 kcal less than the control; however, this value was lower than the minimally clinically significant difference of −115.2 (55). Although when compared to the control, without the brochure, the TLL and PACE labels did not lead to reductions in the selected calories, the addition of an educational brochure led to reductions in the selected calories. The selected calories of the TLL + brochure group were 90 kcal lower than in the TLL group, and in the PACE+brochure they were 55 kcal less than the PACE group. After adjusting for all the confounders, no change was observed in the primary outcome, and, yet, the TLL + brochure group selected the lowest calories.

We may explain why the TLL + brochure combination was more effective than the PACE+brochure one. The first hypothesis is that participants were unfamiliar with the PACE labeling, and although the addition of an educational brochure reduced the amount of selected calories, the reduction was not significant. This hypothesis maybe to some extent reinforced by a study condcuted in poland to identify predictors of food label use (56). They found only one predictor which was self-rated knowledge about nutrition healthiness for label reading. The second probable reason could be that the participants were already familiar with the TLL, but did not know how to use it; indeed, they were guided to a better choice with the help of an educational brochure. The last putative explanation is that the TLL label is simpler than the PACE label, however, more studies are needed to compare the two types of labels. In their study on 172 adults, Blackham et al. assessed the comprehensibility of calorie labels, TLL, and PACE for fast-foods using a questionnaire (57). Consistent with our results, 66% of the participants chose TLL as the most comprehnsible, 18% chose PACE, and 11% chose the calorie label. Two recent studies examined the effects of PACE labels on consumer preferences for healthy and unhealthy foods. In an online survey conducted on 570 Chinese men and women (29), walnut stuffed red dates were considered as the healthy and potato chips as the unhealthy food choices. The participants were randomized into walnut stuffed red dates and potato chips groups, and each group had four choices (1. standard kcal label; 2. a label showing the minutes of walking; 3. running needed to burn off the calories; and 4. a condition without a label). The results indicated that labels enhance the utilization of healthy foods. Moreover, the most positive attitudes were expressed toward the PACE labels rather than the kcal label. One explanation behind the conflicting results of our study and Yang’s may be that their respondents had higher levels of healthy behaviors than the societal average. Elsewhere, another study was conducted on 91 students at a German university to assess the visual attention of PACE labels using eye-tracking technology (28). Participants made choices among 162 snacks –all of which had PACE labels, and 22.91% viewed the PACE label, at least once, for a total mean view time of 3.51 s. However, in the questionnaire administered to the participants, only 8% mentioned noticing the PACE label when purchasing the snacks, and although the general choices they made were healthy, they declared having no specific ‘health’ goal.

Upon comparing the food categories (dairy, sugar-sweetened beverages, cakes, and biscuits) between the study groups, TLL + brochure yeilded the best choices for all the food groups. The maximum difference, which was about 72 kcal, was seen between the TLL + brochure and PACE groups for cakes and biscuits. For dairy products, the largest difference was observed between the TLL + brochure and PACE groups (55 kcal). Finally, in the sugar-sweetened beverages category, the maximum difference was found between the TLL + brochure and TLL groups (19 Kcal).

An earlier study (58) indicated that being female, having a higher educational status and physical activity were associated with higher rates of utilization of nutrition labels, which is consistent with the current study. Based on our findings, individuals with a higher level of education chose fewer calories in all the intervention groups compared to the control. However, in the pairwise comparison of TLL with PACE, and TLL + brochure with the PACE+brochure, for each unit increase in the level of education, higher calories were chosen by the mothers, although it was not statistically significant. Perhaps, more importance should be placed on nutritional knowledge, not on educational level alone. Indeed, results of a review study indicated that nutritional knowledge or nutrition education can have a direct impact on the use of nutrition labels (59).

### 4.3. Policy implications

Based on our findings, it seems that traffic light labels, when accompanied by a brochure, can lead consumers toward food choices with fewer calories.

### 4.4. Limitations

Certain inevitable limitations always exist in qualitative studies, and must be considered when interpreting the findings. Usually, mothers are responsible for the purchase of foodstuff in Iranian households. Therefore, we chose to only interview the schoolchildren’s mothers, and not their fathers, who may have held entirely different opinions. Indeed, with the changing landscape of societal expectations around the roles of males and females, it would be pragmatic to evaluate fathers’ perspectives as well in future research. Additionally, we used a variety of dairy products with familiar brands. We’d recommend examining the impact of the PACE label on both familiar and unfamiliar products in future studies.

We endeavored to consider every viewpoint presented by the mothers when designing the new label. However, certain ideas, such as the use of all-macro fonts, and/or the inclusion of micronutrients in the labels were not feasible. However, we covered more important and comprehensible details regarding the FSN experts’ opinions. In the new label, we simplified our design, by excluding variables such as age and gender. The primary purpose of this study was to design a label which would help people make healthier food choices. We have facilitated this by using a legible font on a colored label that provides consumers with explicit and concise information.

In the quantitative phase, each of the TLL and PACE labels were examined alone and in combination with an educational brochure. To the best of our knowledge, to date, no study has compared PACE and TLL labels in such a way. The main limitation of our study was the one-time exposure to labels, which, particularly in the case of physical activity labels, could have led to its reduced effectiveness as opposed to the TLL. However, the multiplicity of groups, limited time and finances, and difficulty of implementation (lack of school facilities - food spoilage) did not allow us to expose the participants to the labels more than once. Moreover, because the mothers were blind to the purpose of the study, it was practically impossible to expose them several times. Here, the mothers’ education was assessed alone, and their nutritional knowledge was not examined. We therefore recommend evaluating nutritional knowledge in future studies as well. Additionally, it should be noted that physical activity does not eliminate the negative metabolic effects of the food eaten, although our new label also reported higher levels of salt, sugar and fat. Another limitation was that in this study, sampling was done from Tehran. The city accounts for about 10% of the total population of Iran, and due to budget limitations, we could not do it in other cities, but future studies can be done in other provinces as well to ensure the generalizability of results.

Finally, we wanted to design a new PACE label. Though we could not predict what the new label would look like at the beginning of the study, we wanted to make sure it was based on the physical activity information needed to burn calories. This new PACE label also contains information on the amount of sugar/sodium/fat (if present), which is shown in red color and the clause “high in sugar/sodium/fat.” Since both the traffic light label and the new label were colored, we think that the color effect was present in both types of labels.

## 5. Conclusion

The new label presented in this study is a combination of the physical activity, traffic light symbols, and warning labels. According to the mothers in the qualitative phase, the new label helped them make lower caloric choices, even in those with the lowest level of nutritional knowledge. In the quantitative phase, we failed to find any effect of PACE and PACE+brochure on healthier food choices. However, we may arrive at different results if we examine its effect on the selection of restaurant food in future studies, where participants have already been taught about the label. Moreover, according to the findings of this study, training is an important and necessary tool in the utilization of nutrition labels.

## Data availability statement

The raw data supporting the conclusions of this article will be made available by the authors, without undue reservation.

## Ethics statement

The studies involving humans were approved by Research Ethics Committee of Tehran University of Medical Sciences (96-03-161-37037). The studies were conducted in accordance with the local legislation and institutional requirements. The participants provided their written informed consent to participate in this study.

## Author contributions

SS: Conceptualization, Formal analysis, Investigation, Methodology, Project administration, Writing – original draft, Writing – review & editing. SN: Data curation, Formal analysis, Writing – review & editing. ES: Project administration, Writing – review & editing. AD: Project administration, Writing – review & editing. HH: Project administration, Writing – review & editing. AK: Writing – original draft, Writing – review & editing. NA: Project administration, Writing – review & editing. MC: Data curation, Writing – review & editing. CC: Writing – review & editing. AM: Conceptualization, Methodology, Project administration, Supervision, Writing – review & editing.
