# Supplementary material for: Designing a new physical activity calorie equivalent food label and comparing its effect on caloric choices to that of the traffic light label among mothers: a mixed-method study
Source: Front Public Health. 2023 Nov 14;11:1280532. doi: 10.3389/fpubh.2023.1280532 (PMC10683755; doi:10.3389/fpubh.2023.1280532)
Supplement: Supplementary file 1 [file Table_1.DOCX]

| **Supplementary Table 1: General characteristics of mothers participating in the quantitative phase** | |
| --- | --- |
| **Variables** | Mean ± SD or n (%) |
| Age (year) | 38.54 ± 5.00 |
| BMI (kg/m^2^) | 27.63 ± 4.44 |
| **Education** | |
| School graduate | 5 (8.2) |
| High school graduate | 23 (36.1) |
| Associate and bachelor’s degree | 28 (44.0) |
| Master’s and/or higher degree | 7 (11.7) |

| **Supplementary Table 2: Questions asked from the stakeholders** | |
| --- | --- |
| Mothers | 1. What do you think about the PACE labeling? 2. Do you think it might affect your/children’s food choices? 3. Do you think it might affect your/children’s physical activity? 4. What should it look like? 5. What information should it contain? |
| Nutrition and food industry experts | 1. What is your opinion about the newly designed PACE label? 2. What should it look like? 3. What information should it contain? |

**Supplementary Table 3: Mothers' perspectives and expectations and nutrition and food industry experts’ expectations of the new PACE label**

|  | | | | |
| --- | --- | --- | --- | --- |
| **Participants** | **Themes** | **Subthemes** | **Codes** | **Quotes** |
| Mothers | Mothers’ perspectives | Facilitators | Healthier food choices | *“If I see that a product takes an hour to burn off its fat and I have to run for half an hour, I will not take that product at all*.” (31-year-old mother)  *“My husband pays a lot of attention to these things. He usually wants to munch on something, so he reads the calories on the label; desserts and chocolates are considered junk food. He says “oh! There are so many calories in this! He doesn’t eat it at all; he puts it aside. When you know something has many calories, and you have to exert a lot of physical activity you can overcome your craving. This has a great impact on one’s choice, especially for kids. I think that labels would be interesting.”* (39-year-old mother) |
|  |  |  | Easy to understand | *“Some people may not understand the meaning of 18 calories, as calories are not known by many; it is an unfamiliar concept. Something clearer would be better; for example, something that tells you how much you should walk to burn the calories. Take for example crisps, if it’s written 500 calories, the person may not understand what it means. S/he doesn’t know it may be equivalent to walking 2-3 hours”* (40-year-old mother) |
|  |  |  | Suitable solution | *“People continue to eat certain unhealthy foods that are high in sugar or fat even when they know they're harmful. However, when you give a solution, e.g. you say a* gaz *(a local sweet) has these many calories, and the way to burn its calories is to walk for an hour or vacuum-clean then it makes sense”* (44-year-old mother) |
|  |  |  | Motivation to increase physical activity | *“In my opinion, the physical activity required to burn off the calories of let’s say a cake, can become a habit. So, after eating a cake or an ice-cream, I will try to burn the calories.”* (41-year-old mother) |
|  |  | Barriers | Economic problems | *“When a mother is only concerned with feeding her child, she doesn’t care about the nutrition label. We should keep these mothers in mind too.”* (39-year-old mother) *“Someone in this economic situation does not look at the fat content of a product; she just wants to feed herself and her family.”* (41-year- old mother) |
|  |  |  | Lack of time | *“We have so many problems with our children. When I feel hungry, I eat something and go back to help them with their lessons. I do not have time to burn calories.”* (30-year-old mother) |
|  |  |  | Impact on level of activity | *“Physical activity is good for those who exercise; personally, I am not a sportsperson. I can’t do the walking or exercise part. I only went for two weeks in the summer and that was that!”* (41-year-old mother)  On the contrary, another mother had a completely different opinion and believed lack of physical activity stemmed from habits.  *“I think that anyone can dedicate an hour a day to exercise and walking. Even employed people can set aside half an hour for walking. It’s mostly because of habits, not a busy lifestyle.”*(39-year-old mother) |
|  |  |  | Lack of significance |  |
|  |  | Appearance | Graphical features | *“I think physical activity complements the TLL. I mean that if both of them are put together, it will be complete. They both have information about fat, sugar, carbohydrates, caloric content, and physical activity, which is sufficient.”* **(Supp. Fig. 1**) |
|  |  |  | Colorful |  |
|  |  |  | Front-of-package |  |
|  |  |  | Legible |  |
|  |  | Information | Calorie content |  |
|  |  |  | Fat |  |
|  |  |  | Sugar |  |
|  |  |  | Salt |  |
|  |  |  | Vitamins and minerals |  |
|  |  |  | Understandable portion size |  |
|  |  |  | Minutes of physical activity (walking) |  |
| Nutrition and food industry experts | Nutrition and food industry experts' expectations of the new PACE label | Appearance | Graphical features | *“If I were to declare a product as unhealthy based on the TLL colors, I would have used one single color. I would advise against the use of three colors.”* |
|  |  |  | Single colored |  |
|  |  | Information | Concise information | *“In my opinion, the calorie content must be included. Fat, be it good or bad, is also an important factor as it results in an intake of calories. I believe, considering our society’s level of nutrition knowledge, the information that is currently being provided about calories, salt and fat is sufficient.”* |
|  |  |  | Highlighting the high amount of fat, sugar and salt |  |
| PACE: physical activity calorie equivalent; TLL: traffic light label | | | | |

| Supplementary Table 4a: Color guide based on Iran's Food and Drug Administration guidelines for solid foods |
| --- |

| Value | Small | Moderate | High | |
| --- | --- | --- | --- | --- |
| Color marker | Green | Orange | Red | Red |
| Indicator | In 100 grams of product | In 100 grams of product | In 100 grams of product | In one serving of product |
| Sugar | ≤ 5 grams | >5 to ≤ 22.5 grams | >22.5 grams | > 27 grams |
| Total fat | ≤ 3 grams | >3 to ≤ 17.5 grams | >17.5 grams | > 21 grams |
| Salt (sodium chloride) | ≤ 0.3 gram | >0.3 to ≤ 1.5 grams | >1.5 grams | > 1.8 grams |
| Trans fatty acids | ≤ 0.5 gram | >0.5 to ≤ 2grams | >2 grams | > 2 grams |

| Supplementary Table 4b. Color guide based on the Iran's Food and Drug Administration guidelines for the liquid food |
| --- |

| Value | Small | Moderate | High | |
| --- | --- | --- | --- | --- |
| Color marker | Green | Orange | Red | Red |
| Indicator | In 100 ml of product | In 100 ml of product | In 100 ml of product | In one serving of product |
| Sugar | ≤ 2.5 grams | >2.5 to ≤ 11.25 grams | >11.25 grams | > 13.5 grams |
| Total fat | ≤ 1.5 grams | >1.5 to ≤ 8.75 grams | >8.75 grams | > 10.5 grams |
| Salt (sodium chloride) | ≤ 0.3 gram | >0.3 to ≤ 0.75 grams | >0.75 grams | > 0.9 grams |
| Trans fatty acids | ≤ 0.5 gram | >0.5 to ≤ 2grams | >2 grams | > 2 grams |

| **Supplementary Table 5: Comparing the mean calories of selected foods in TLL, TLL+ brochure, PACE, and PACE + brochure to the control after adjusting for confounders^1^** | | | |
| --- | --- | --- | --- |
| **Comparisons** | | **β Coefficients (95%CI)** | **P Value** |
| **Intervention groups** | **Control** | **Reference** |  |
|  | TLL | 4.93 (-55.40, 65.28) | 0.87 |
|  | TLL + Brochure | -75.54 (-136.76, -14.31) | 0.01 |
|  | PACE | 60.90 (0.157, 121.65) | 0.04 |
|  | PACE + Brochure | 9.66 (-51.40, 70.74) | 0.75 |
| **Mother’s age (years)** | | -1.41 (-3.93, 1.10) | 0.27 |
| **Economic status based on assets** | | -1.99 (-15.29, 11.30) | 0.76 |
| **Education level (academic/ non-academic)** | | -9.14 (-67.79, 49.50) | 0.75 |
| **Occupation (employed, housewife)** | | 37.62 (-10.72, 85.98) | 0.12 |
| **BMI** | | | |
| **Normal** | | **Reference** |  |
| **Overweight** | | 17.49 (-26.70, 61.70) | 0.43 |
| **Obese** | | 104.45 (55.01, 153.89) | 0.0001 |
| **Physical activity** | | -12.95 (-49.27, 23.37) | 0.48 |
| ^1^The confounders are age, economic status, education, occupation, BMI, and physical activity  ^2^Assets were categorized into quintiles from the lowest to the highest (The reference category is the lowest economic status)  ^3^The reference category is non-academic education  ^4^Physical activity was categorized into tertiles (light, moderate, vigorous)  ^5^Multivariable regression was used to compare the effect of the PACE label with TLL on the calories of selected foods  PACE: physical activity calorie equivalent; TLL: traffic light label | | | |

| **Supplementary Table 6: Comparing the effect of PACE + brochure and TLL+ brochure on calories of the selected foods –bearing in mind the confounders’^1^ effects** | | |
| --- | --- | --- |
| **Comparisons** | **β Coefficients (95% CI)** | **p Value** |
| **Calorie intake (kcal, PACE + brochure /TLL+ brochure)** | 40.08 (10.96 - 69.20) | 0.007 |
| **Mothers’ Age (years)** | -2.12 (-4.93 - 0.69) | 0.13 |
| **Economic status based on assets^2^** | -0.67 (-20.19 - 96.62) | 0.94 |
| **Education level (academic/ non-academic)^3^** | 40.49 (-74.12 - 155.10) | 0.48 |
| **Occupation (employed, housewife)** | 33.79 (-43.94 - 111.53) | 0.39 |
| **BMI^4^** |  |  |
| **Normal** | **Reference** |  |
| **Overweight** | 63.66 (-2.72, 130.06) | 0.06 |
| **Obese** | 138.36 (60.07, 216.66) | 0.001 |
| **Physical activity ^5^** | -28.52 (-79.21, 22.17) | 0.26 |
| ^1^The confounders are age, economic status, education, occupation, BMI, and physical activity  ^2^Assets were categorized into quintiles from lowest to the highest (The reference category is the lowest economic status)  ^3^The reference category is non-academic education  ^4^Physical activity was categorized into tertiles (light, moderate, vigorous)  ^5^Multivariable regression was used to compare the effect of the PACE label with TLL on the calories of selected foods  PACE: physical activity calorie equivalent; TLL: traffic light label | | |

| **Supplementary Table 7: Mean time taken for the selection of food products for each group** | | | | | | |
| --- | --- | --- | --- | --- | --- | --- |
|  | Without label | TLL | TLL+ brochure | PACE | PACE + brochure | P value |
| Participants (number) | 97 | 100 | 99 | 98 | 99 |  |
| Time ^a^ (min) | 1.69  (0.71) | 1.82  (1.16) | 2.16  (2.21) | 1.72  (1.05) | 1.76  (0.93) | 0.089 |
| ^a^ The values are means (SD)  ^b^ p value obtained from the ANOVA test  PACE: physical activity calorie equivalent; TLL: traffic light label | | | | | | |
