# Supplementary material for: Designing a new physical activity calorie equivalent food label and comparing its effect on caloric choices to that of the traffic light label among mothers: a mixed-method study
Source: Front Public Health. 2023 Nov 14;11:1280532. doi: 10.3389/fpubh.2023.1280532 (PMC10683755; doi:10.3389/fpubh.2023.1280532)
Supplement: Supplementary file 2 [file Table_2.DOC]

.

**Do you know what key points are when choosing food products?**

The traffic light label has 5 indicators to help you make better food choices.

There are five indicators in the color indicators: 1.Energy, 2. Sugar, 3. Fat, 4. Salt, 5. Trans fatty acids

Green means ‘low’. For example, if the sugar is green in a product, it means that the amount of sugar in that food product is low.

Orange means ‘medium’. For example, if the fat in a product is orange, it means that the amount of fat is moderate and you should be careful when buying it.

Red indicates ‘large’ amounts. For example, if the salt in a product is red, it means that the amount of salt in that product is high, and it is better for a person with high blood pressure to choose another product that is low in salt.


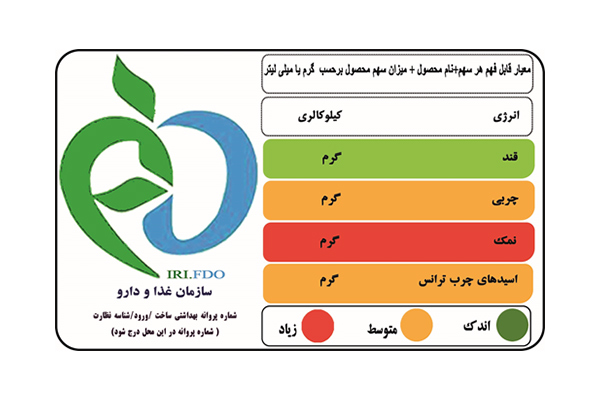


This food label shows the amount of energy and duration of brisk walk (in minutes) required to burn off the amount of calories in a product. If there is a high amount of sugar or fat or salt, it is shown in red, which comes as a warning to you. This label allows you to make healthier choices based on your (family’s) physical condition.

Green means that the amount of sugar, fat and salt is moderate or low.
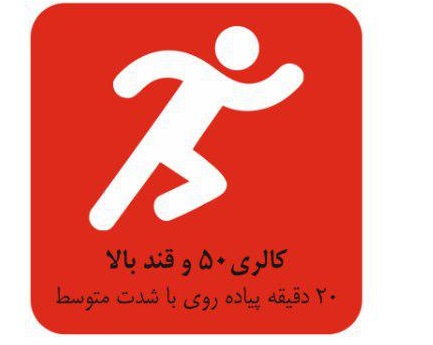
**
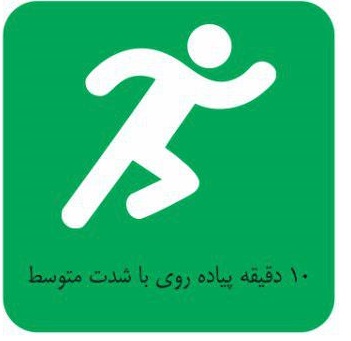
**


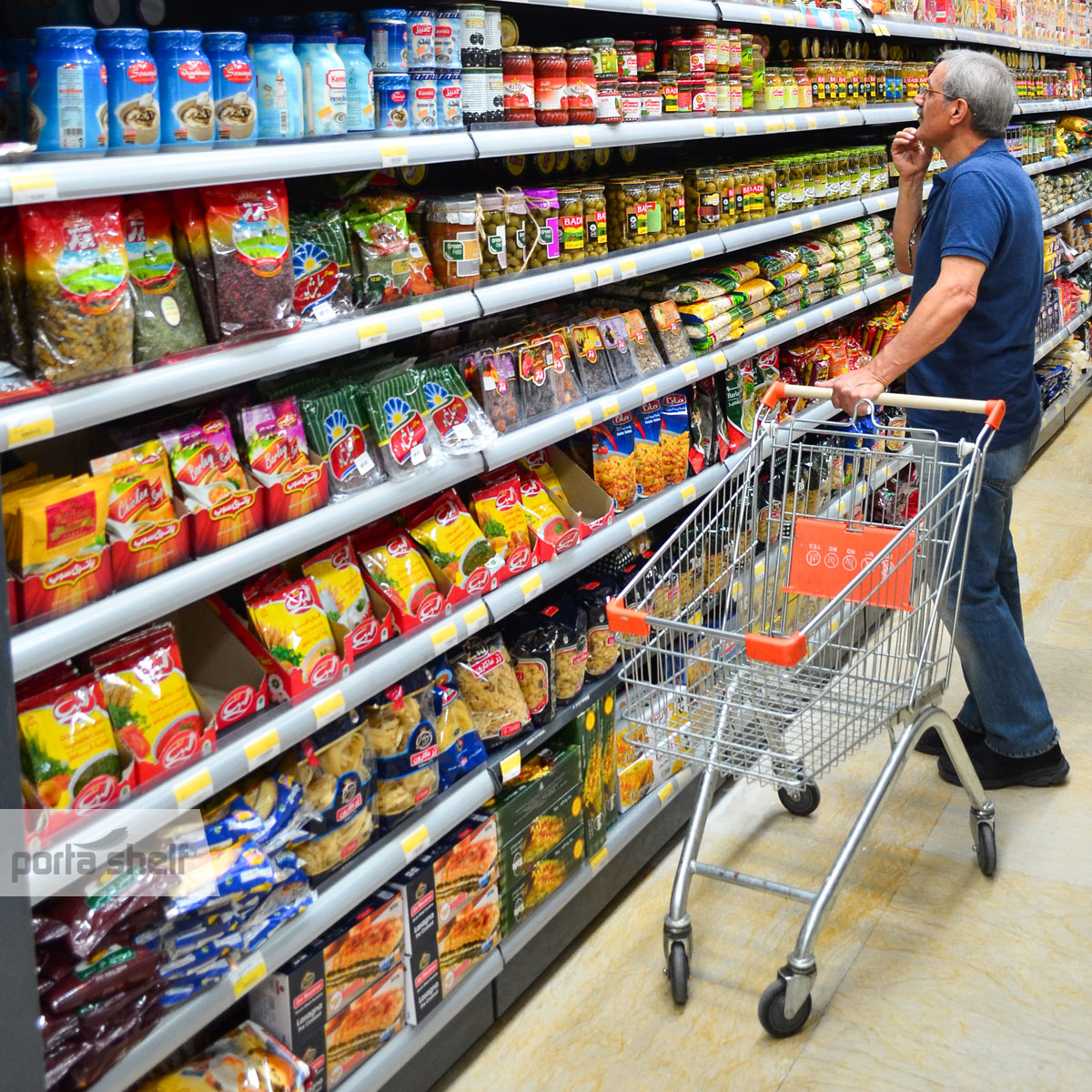


The manufacturing license number and standard sign, ingredients, additives and preservatives are all important. Attention must also be paid to the product’s address and place of production; if they have not been listed on the product, it could indicate that the product is fake.


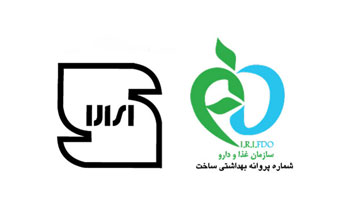


It is mandatory to include the production and expiration dates on all food products, and this should be considered when choosing any product. This holds especially true for products that are highly perishable, such as milk.


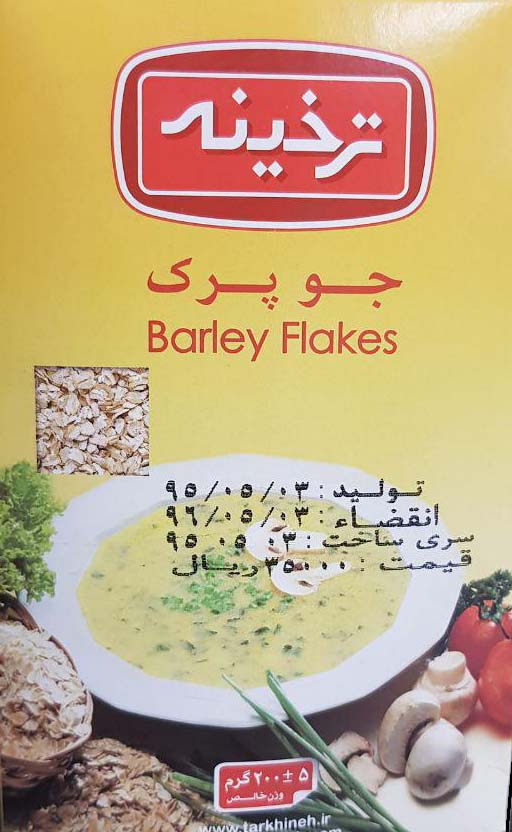


The product packaging should not be torn. Tears or holes in the packaging can be entrance points for microbial contamination.

**
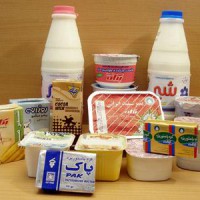
**
