# Supplementary material for: Designing a new physical activity calorie equivalent food label and comparing its effect on caloric choices to that of the traffic light label among mothers: a mixed-method study
Source: Front Public Health. 2023 Nov 14;11:1280532. doi: 10.3389/fpubh.2023.1280532 (PMC10683755; doi:10.3389/fpubh.2023.1280532)
Supplement: Supplementary file 3 [file Table_3.DOCX]

| Address: ………….……………  Phone number: ………………… | **Project code approved by Tehran University of Medical Sciences:** 96-03-161-37037  بسمه تعالي **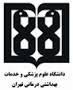**  **Title of project:** Investigating the effect of traffic light labeling and physical activity calorie equivalent labeling on food choices | |
| --- | --- | --- |
|  |  |  |
|  |  |  |
|  | All information will remain confidential and will not be shared with others. The researchers will investigate and conclude the information collectively. | |
| 1. Identification number | |  |
| 1. Number of family members 88. Do not want to answer | |  |
| 1. Number of household members (number of people at one table)   88. Do not want to answer | |  |
| 1. Ethnicity: 1) Turk 2) Fars 3) Kurd 4) Baloch 5) Northern 6) Other 8. Do not want to answer 9) Do not know | |  |
| 1. The speed of eating lunch in minutes   888. Do not want to answer  . | |  |
| 1. Marital status 1) Married 2) Widow 3) Divorced | |  |
| 1. Occupational status (mother) 2. Housekeeper 3. Worker (Construction - Factory - Services) 4. Farmer 5. Office clerk 6. Management employee (doctor, lawyer, manager, faculty- ...) 7. Retired 8. Self-employed 9. Do not want to answer | |  |
| Level of education   1. Illiterate or reading and writing literacy 2. High School 3. High school graduate 4. Associate degree 5. **Bachelor’s degree** 6. **Master’s degree** 7. **Doctoral degree** 8. **Do not want to answer** | |  |
| 11. Which of the following items do you have?  1) Side by Side Freezer Refrigerator  2) Large LCD TV  3) Automatic washing machine  4) Dish washing machine  5) Handmade carpet  6) Personal computer and netbook  7) Car (If yes, its current price)  8) Microwave  9) Classic style sofas  10) Rechargeable vacuum cleaner  11) DVD | |  |
| 19. Do you feel hungry at the moment?  1) Yes 2) No | | |
| 20.. Which of these best describes your current state of appetite?  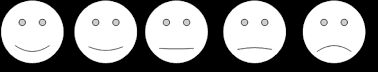   - **Neither hungry nor full**   **Almost full**  **Full**  **Hungry**  **Almost hungry** | | |
| 20. Birth date | | .... ..../.... .... /..... ..... |
| 21. Weight (kg) | | .........**/** ........ ......... |
| 22. Height (m) | | ......... ........**/** ......... |
